# Novel ellipsoid-like granules exhibit enhanced anammox performance compared to sphere-like granules

**DOI:** 10.1016/j.wroa.2024.100270

**Published:** 2024-10-30

**Authors:** Dongdong Xu, Tao Liu, Chao Pan, Leiyan Guo, Jianhua Guo, Ping Zheng, Meng Zhang

**Affiliations:** a Department of Environmental Engineering, College of Environmental & Resource Sciences, Zhejiang University, Hangzhou, China; b Australian Centre for Water and Environmental Biotechnology (ACWEB, formerly AWMC), The University of Queensland, St. Lucia, Queensland 4072, Australia; c Zhejiang Province Key Laboratory for Water Pollution Control and Environmental Safety, Hangzhou, China; d Innovation Center of Yangtze River Delta, Zhejiang University, Jiashan 314100, China

**Keywords:** Anammox granular sludge, Flow shear, Granular morphology, Shape, Mass transfer, Anammox activity

## Abstract

Anammox granular sludge (AnGS) serves as an important platform for cost-effective nitrogen removal from wastewater. Different to the traditionally sphere-like granules, a novel type of AnGS in a unique ellipsoid-like shape was obtained through enhancing shear force. The ellipsoid-like AnGS significantly exhibited a smaller aspect ratio (-25.1 %) and granular size (-11.8 %), compared to traditional sphere-like AnGS (*p* < 0.01). Comprehensive comparisons showed that ellipsoid-like AnGS possessed a significantly higher extracellular polymeric substances (EPS) content and strength, as well as an enhanced mass transfer and a higher viable bacteria proportion due to the larger substrate permeable zone (*p* < 0.01). Additionally, the anammox bacterial abundance (*Candidatus* Kuenenia) was 12.2 % higher in ellipsoid-like AnGS than in sphere-like AnGS. All these characteristics of ellipsoid-like AnGS jointly increased the specific anammox activity by 29.0 % and nitrogen removal capacity by 22.6 %, compared to sphere-like AnGS. Further fluid field simulation suggested the enhanced flow shear on the side surface of AnGS likely drove the formation of ellipsoid-like AnGS. The higher shear force on the side surface led to an increase of EPS content (especially hydrophobic protein) and elastic modulus, thus constraining lateral expansion. This study sheds light on impacts of granular shape, an overlooked morphological factor, on anammox performance. The ellipsoid-like AnGS presented herein also offers a unique and promising aggregate to enhance anammox performance.

## Highlights

• Enhanced flow shear shaped ellipsoid-like AnGS (eAnGS).
• The impacts of different granule shapes were systematically investigated.
• eAnGS had higher SAA and maximal NRR compared to sphere-like AnGS.
• eAnGS improved the mass transfer of AnGS by holding a larger permeable zone.
• The formation of eAnGS could be attributed to fluid action and bacterial response.

## Graphical abstract

**Figure:**
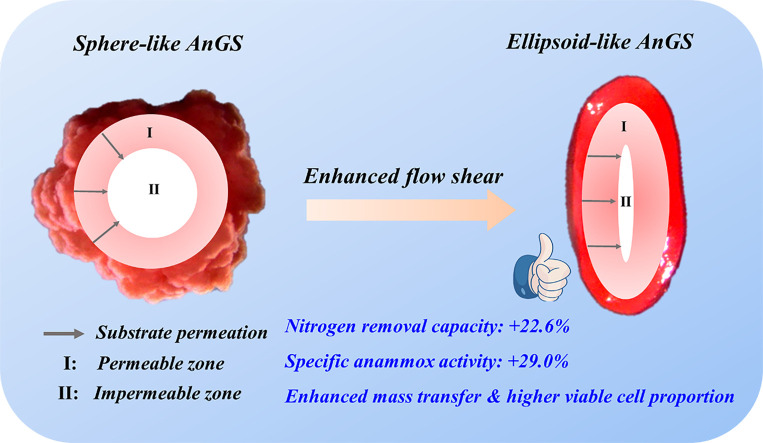

## 1 Introduction

Anammox has been recognized as a sustainable process for biological nitrogen removal mediated by anammox bacteria (Kartal et al., 2010). Compared with conventional nitrification-denitrification processes, anammox process presents distinct advantages, including no organics demand, low energy consumption, and minimal sludge production (Kartal et al., 2011; Zhang et al., 2019). Currently, anammox-based biotechnologies have attracted worldwide attention for treating both high-strength and municipal wastewaters (Weralupitiya et al., 2021; Zhang et al., 2021a). Among all different configurations, anammox granular sludge (AnGS) is a popular platform for their applications (Lotti et al., 2014; Zhang et al., 2022; Xue et al., 2023), given that AnGS can effectively retain microorganisms and enhance the robustness against varying environmental conditions (Wong et al., 2023).

Granular size has been considered as the most critical parameter affecting the performance of AnGS (Volcke et al., 2012). While earlier studies primarily focused on formation of AnGS and obtaining large granules for better biomass retention, recent studies have realized the impacts of size might be more complicated (Song et al., 2023). For instance, Zhu et al. (2018) showed the decreased activity of AnGS in small (<0.5 mm) and large granules (>0.9 mm). Liu et al. (2017) demonstrated the difficulty in maintaining the nitrogen removal in AnGS with a size of <0.2 and >1.0 mm. In nearly all previous studies focusing on AnGS, the granular shape was observed or assumed to be spherical, thus the size measurements were primarily in one dimension. However, such assumptions may oversimplify complexity of granule morphology and neglect the impacts of different granular shapes on nitrogen removal performance.

Indeed, different shapes have been observed across different types of granules. Chen et al. (2017) showed that the proportion of ellipsoidal aerobic granules increased along with the elevation of wastewater loading rates. Baloch et al. (2006) demonstrated that the anaerobic granules cultivated in a full-scale up-flow anaerobic sludge blanket exhibited two different dimensions along two axes, very different from typical spherical granules. Li et al. (2014) also found the formation of streamlined denitrifying granules with a better settleability. Despite these observations, whether different granular shapes result in different sludge properties such as EPS content and strength, and more importantly, the impacts of different granular shapes on process performance remain elusive.

Herein, the relationships between the morphology (shape and size) of AnGS and anammox activity were systematically studied. In detail, two reactors inoculated with traditional sphere-like AnGS were operated for over 220 days. Assuming the granular shape is mainly affected by hydraulics, the experimental group was operated with external dinitrogen gas supply to enhance shear force and to form AnGS in a novel ellipsoid-like shape. We systematically compared the nitrogen removal performance, specific anammox activity (SAA), mass transfer capacity, EPS properties, and microbial community in traditional sphere-like and novel ellipsoid-like AnGS. Additionally, the study also simulated the fluid field to reveal the shaping and formation of the novel ellipsoid-like AnGS by flow shear.

## 2 Results

### 2.1 Enhanced nitrogen removal capacity with higher flow shear

**Fig. 1:**
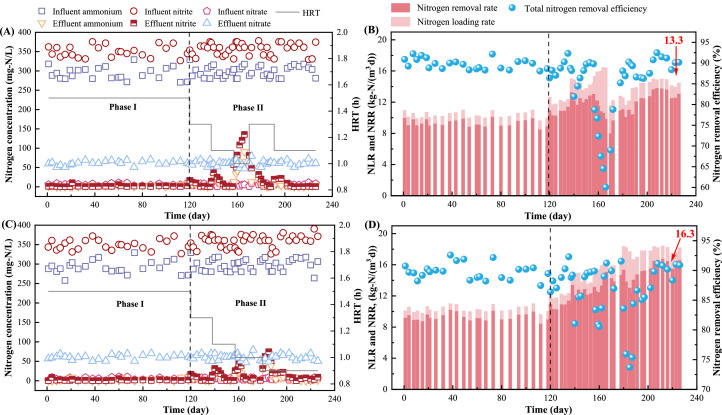
Operation performance of R_CK_ and R_GS_. (A and B referred to concentrations of various nitrogen species and HRT, NLR, NRR and NRE of R_CK_; C and D referred to the corresponding profiles of R_GS_).

Two anammox reactors (R_CK_ and R_GS_) without and with the external gas supply were operated for 226 days. In phase I (days 1–119), the NLR remained stable with constant influent total nitrogen (TN) concentrations and HRT, while in phase II (days 120–226), the HRT was step-wise shortened to elevate NLR (Fig. 1). In detail, during phase I, both R_CK_ and R_GS_ showed efficient performance. The nitrogen removal efficiency (NRE) and NRR of R_CK_ and R_GS_ were 89.8 ± 1.3 % and 89.4 ± 1.7 %, 9.4 ± 0.4 and 9.3 ± 0.5 kg-N/(m^3^·d), respectively. Notably, the effluent suspended solids concentration of R_GS_ rose sharply on day 100 as the external gas rate was adjusted upwards to 1.33 cm/min, indicating the biomass washout. Thus, the external gas rate was decreased to 1.00 cm/min in the following operation.

During phase II, the HRT was gradually decreased to explore nitrogen removal capacities. Apparently, a transient rose and then a gradual decline in effluent ammonium and nitrite concentrations was observed in both reactors when the HRT was shortened from 1.5 to 1.3 h on day 120, and further to 1.1 h on day 138. However, as HRT was shortened to 1.0 h on day 157, R_CK_ performance deteriorated quickly with NRE decreasing to 60.1 %, implying R_CK_ reached capacity upper limit. The HRT of R_CK_ was changed to 1.3 h on day 170 and to 1.1 h on day 191, leading to progressively recovered NRR of 13.3 ± 0.6 kg-N/(m^3^·d) (Fig. 1A and B). In contrast, R_GS_ exhibited minor fluctuation in the effluent TN concentration when the HRT was shortened to 1.0 h on day 138. Thus, the HRT was further shortened to 0.9 h on day 170. Despite the transient increase in effluent ammonium and nitrite, after 7 days adaption, the effluent TN concentration declined to 65.0 ± 6.3 mg-N/L with stable NRR of 16.3 ± 0.5 kg-N/(m^3^·d). These collectively demonstrated R_GS_ with higher flow shear exhibited an enhanced nitrogen removal capacity, which showed an elevation of NRR by 22.6 % than R_CK_ (Fig. 1C and D).

### 2.2 Distinct morphology of AnGS in two systems

**Fig. 2:**
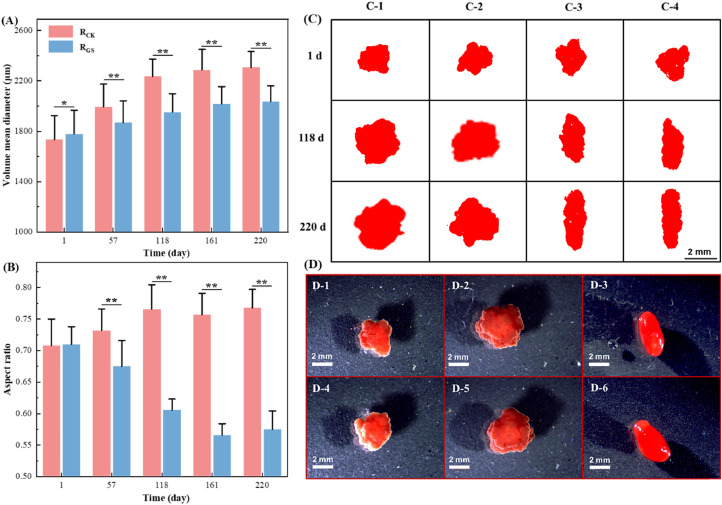
Size (A) and shape (B) of AnGS indicated by VMD and aspect ratio (more than 10^3^ granules were determined in each test). Laser projection (C-1&2 and C-3&4 referred to AnGS in R_CK_ and R_GS_, respectively) and morphology (D-1&4 referred to seeding AnGS; D-2&5 and 3&6 referred to AnGS in R_CK_ and R_GS_ on day 220, respectively) of AnGS in R_CK_ and R_GS_ during operation.

Size and shape are two key morphological parameters of AnGS (Song et al., 2023), which can be described by volume mean diameter (VMD) and aspect ratio, respectively. The VMD of AnGS in R_CK_ and R_GS_ both displayed an increasing trend (Fig. 2A), with values rising from 1734 and 1778 μm on day 1 to 2306 and 2035 μm on day 220, respectively. Notably, the VMD of AnGS in R_GS_ was 13.3 % lower than that in R_CK_ on day 220. Specifically, for R_CK_, the proportion of AnGS in size fraction of 2000–3000 μm rose substantially and gradually became the dominant size fraction. While the corresponding increment in R_GS_ was much lower (Fig. S1). Moreover, the aspect ratio of AnGS in R_GS_ decreased sharply from 0.71 ± 0.03 on day 1 to 0.57 ± 0.02 (*p* < 0.05), while that of R_CK_ rose from 0.71 ± 0.04 to a stable level of 0.77 ± 0.04 (Fig. 2B). These demonstrated AnGS in R_GS_ was morphologically different from that in R_CK_. Observations revealed that projection area of AnGS in R_CK_ extended gradually in all dimensions, ultimately forming a circular shape. In contrast, granules in R_GS_ mainly extended along one dimension, resulting in an ellipsoid-like shape (Fig. 2C), consistent with the results of the aspect ratio. Stereoscopic observation further confirmed ellipsoid-like granular shape in R_GS_ (Fig. 2D). Thus, the strong shear shaped ellipsoid-like AnGS with a smaller size, and the aspect ratio can be applied as a parameter to distinguish sphere-like and ellipsoid-like AnGS.

### 2.3 Different characteristics of sphere-like and ellipsoid-like AnGS

#### 2.3.1 Ellipsoid-like AnGS showed higher SAA

**Fig. 3:**
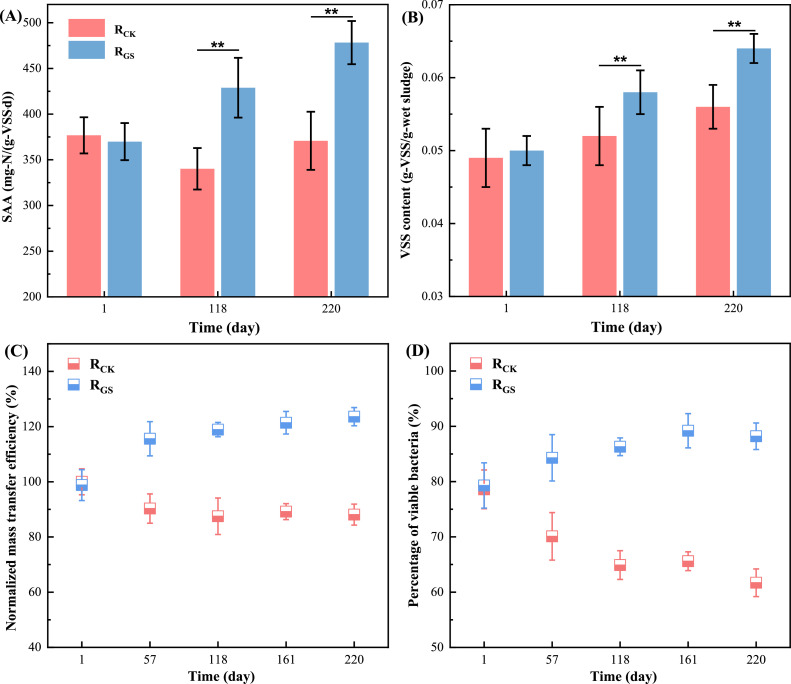
SAA (A), VSS content (B), normalized mass transfer efficiency (C) and ratio of viable bacteria (D) of AnGS in R_CK_ and R_GS_ during operation. (Mass transfer efficiency was normalized based on data of R_CK_ on day 1).

The initial SAA of AnGS in R_CK_ and R_GS_ showed no significant difference (*p*>0.05) (Fig. 3A). For R_CK_, the SAA presented its stability during most of operation, but showed a decrease at the end of phase I. For R_GS_, the SAA of AnGS displayed an increase from 428.9 ± 32.7 mg-N/(g-VSS·d) on day 180 to 478.3 ± 23.6 mg-N/(g-VSS·d) on day 220, which was 26.1 % and 29.0 % higher than those of AnGS in R_CK_. The initial VSS of AnGS was similar in R_CK_ and R_GS_, in which VSS rose constantly during operation, but the increase rate of R_GS_ was faster. On day 220, the VSS of AnGS in R_GS_ was 14.3 % higher than that in R_CK_ (Fig. 3B). However, the sludge concentration of R_GS_ declined slightly due to the increase of sludge bed expansion ratio with the external gas supply. On the whole, the VSS concentrations of two reactors were comparable (*p*>0.05, Fig. S2). Therefore, the enhanced performance of R_GS_ was mainly ascribed to the improved SAA of ellipsoid-like AnGS.

#### 2.3.2 Ellipsoid-like AnGS showed enhanced mass transfer capacity

The SAA of AnGS is positively related to mass transfer (Shi et al., 2017). Mass transfer efficiency *η*, defined as the ratio of actual reaction rate to the maximum reaction rate, is applied to characterize mass transfer capacity of AnGS (Xu et al., 2021a). A higher *η* value indicates a better mass transfer capacity. The initial *η* of AnGS in R_CK_ and R_GS_ were similar (Fig. 3C). For R_CK_, the *η* decreased slightly during the whole operation, while for R_GS_, the *η* showed an obvious upward trend. On day 220, the *η* of AnGS in R_GS_ was 35.5 % higher than that in R_CK_. Similarly, the viable bacterial ratio in R_CK_ declined obviously during the whole operation while the counterparts in R_GS_ rose slightly (Fig. 3D). Generally, the larger viable bacterial ratio implies the better mass transfer. Collectively, ellipsoid-like AnGS in R_GS_ showed a better mass transfer, thus promoting SAA and enhancing NRR.

#### 2.3.3 Ellipsoid-like AnGS possessed higher EPS content and strength

**Fig. 4:**
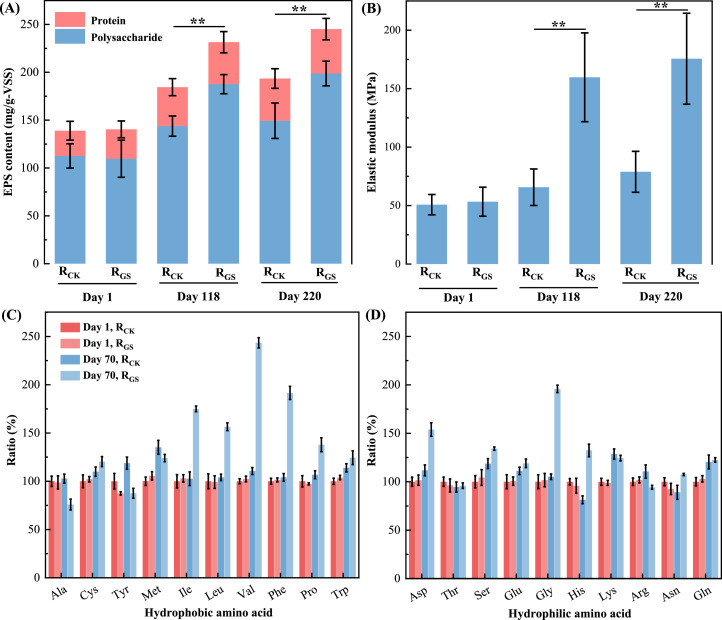
EPS content (A), elasticity modulus (B) of AnGS in R_CK_ and R_GS_ during operation; The abundance of hydrophobic (C) and hydrophilic (D) amino acids. (The full names of amino acids were shown in Table S1; The amino acid's abundance in C and D were normalized based on data of R_CK_ on day 1).

EPS present a three-dimensional network structure and exert a great influence on granular morphology (Flemming and Wingender, 2010; Hou et al., 2015). The EPS content of both R_CK_ and R_GS_ increased, while the increment of EPS content in R_GS_ was significantly higher than that in R_CK_ (*p* < 0.01), especially the protein content (Fig. 4A). Elasticity modulus can reflect the EPS strength, with a larger elastic modulus indicative of greater EPS strength (Jorba et al., 2017; Wang et al., 2022). The surface elastic modulus of AnGS in both R_CK_ and R_GS_ showed an overall increase trend, while the increment of R_GS_ was significantly larger than that of R_CK_ (*p* < 0.01) (Fig. 4B), indicating the higher EPS strength of R_GS_, which agreed with EPS determinations, showing a positive relationship between EPS contents and granular elastic modulus. Additionally, the surface elastic modulus of AnGS in R_GS_ was more heterogeneous, with significantly smaller values along the long axis of ellipsoid-like AnGS (*p* < 0.05) (Fig. S3). Also, EPS content at the two ends of the long axis of the ellipsoid-like AnGS was relatively less compared to the other sides (Fig. S4).

Amino acids analysis suggested that contents of hydrophobic amino acids, such as phenylalanine (Phe), valine (Val), and leucine (Leu) in the protein of AnGS in R_GS_ were higher than those of AnGS in R_CK_ (*p* < 0.05) (Fig. S5). Additionally, we investigated bacterial amino acids metabolism by untargeted metabolomic analysis. In the initial state, no significant difference was detected in the amino acids’ abundance between R_CK_ and R_GS_. Most of amino acids abundance rose in R_CK_ and R_GS_ on day 70, while the increment in R_GS_ was higher (*p* < 0.05), especially for aspartate (Asp), glycine (Gly), valine (Val), isoleucine (Iso), leucine (Leu) and phenylalanine (Phe) (Fig. 4C and D). Notably, these amino acids with dramatic increase were hydrophobic, suggesting flow shear promoted biosynthesis of hydrophobic amino acids, leading to the increase of hydrophobic protein in EPS (Fig. 4A).

### 2.4 Microbial communities in sphere-like and ellipsoid-like AnGS

*Candidatus* Kuenenia, *Denitratisoma*, A4b_norank, SBR1031_norank were detected as the main genera, accounting for over 60 % of the microbial community (Fig. S6). Initially, the microbial compositions of sphere-like and ellipsoid-like AnGS were similar. For sphere-like AnGS, the abundance of *Candidatus* Kuenenia rose from 40.3 % to 47.2 %, while *Denitratisoma* belonging to denitrifying bacteria remained stable during the operation. Heterotrophic A4b_norank and SBR1031_norank, which utilize dead bacteria and cell secretion, rose from 6.5 % and 5.3 % on day 1 to 11.9 % and 11.7 % on day 220, respectively. For ellipsoid-like AnGS, the abundance of *Candidatus* Kuenenia and *Denitratisoma* rose significantly from 38.9 % and 10.9 % on day 1 to 59.4 % and 15.7 % on day 220, respectively, while A4b_norank and SBR1031_norank kept stable. These results demonstrated anammox bacterial abundance in ellipsoid-like AnGS was higher than that in sphere-like AnGS.

### 2.5 Flow field shaped the ellipsoid-like AnGS

**Fig. 5:**
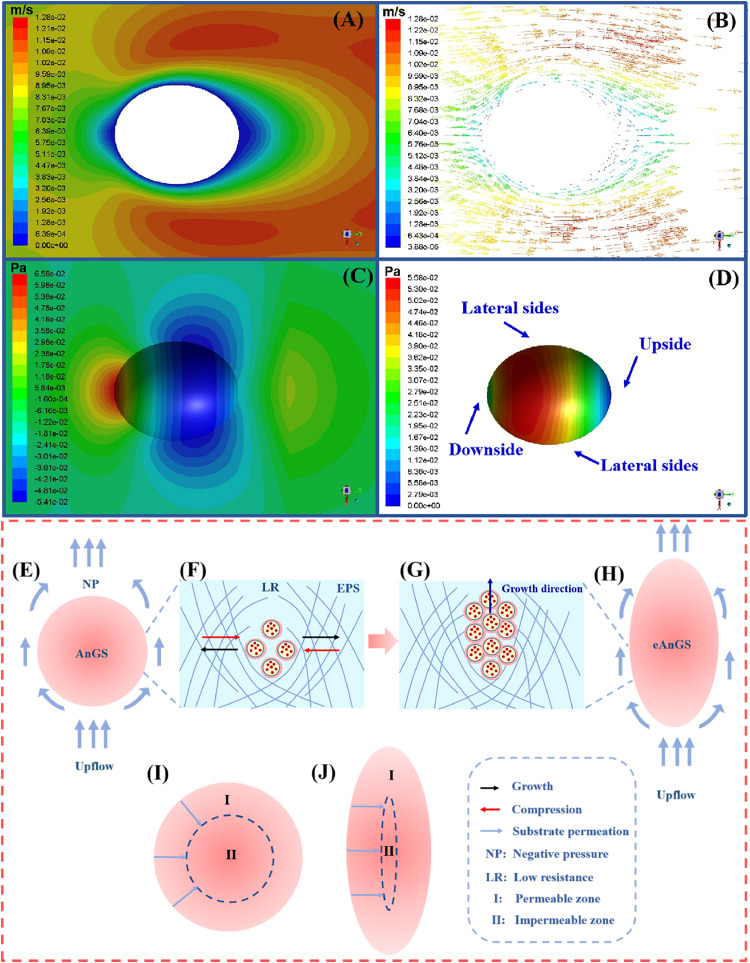
Simulation of flow field (A), flow line (B), the hydraulic pressure (C), and shear force (D) with a simplified model. The flow direction from the left to the right referred to the flow from the bottom to the top in the reactor. Growth of anammox bacteria in AnGS under enhanced shear force (E, F, G and H), and the substrate permeable zone in sphere-like and ellipsoid-like AnGS (I, J). (eAnGS referred to ellipsoid-like AnGS).

The morphology of AnGS is closely related to the surrounding flow field. To reveal the formation mechanism of ellipsoid-like AnGS, the flow field surrounding AnGS in R_GS_ was stimulated. The fluid exhibited a bottom-to-top flow pattern within the bioreactor, with the upward flow firstly contacting the downside of AnGS and then moving away from the upside of AnGS (Fig. 5A and B). The hydraulic pressure and shear force exerted on the upside of AnGS were lower than those on the downside and lateral sides (Fig. 5C and D). Additionally, the shear force applied on the lateral sides of AnGS was notably greater than on other sides, potentially resulting in thinning of the lateral aspects (Fig. 5D). Overall, the surface pressure exerted on AnGS would increase, and more importantly, the discrepancy between the lateral sides and the two ends became considerably more pronounced under heightened flow shear induced by the external gas supply, thus intensifying the shaping effect on AnGS.

## 3 Discussion

### 3.1 A novel ellipsoid-like AnGS shaped by the enhanced flow shear

Through enhancing flow shear by providing external gas, we obtained a novel type of ellipsoid-like AnGS. The size and aspect ratio of ellipsoid-like AnGS were 11.8 % and 25.1 % less than the traditional sphere-like AnGS, respectively. The ellipsoid-like AnGS also possessed elevated levels of EPS content and greater strength, indicative of their enhanced structural stability. Enhancing flow shear by elevating the liquid/gas up-flow can promote the microbial EPS secretion (Liu and Tay, 2002; Manonmani and Joseph, 2018). Building upon this understanding, we found bacterial synthesis of hydrophobic amino acids rose largely at enhanced flow shear, while the EPS content, especially hydrophobic proteins, rose accordingly, which may contribute to the larger surface elasticity modulus of ellipsoid-like AnGS.

In AnGS, anammox bacteria are confined into the three-dimensional network of EPS (Wang et al., 2020). EPS strength exerts great effects on bacterial movement and growth (Even et al., 2017; Chaudhuri et al., 2020; Dufrene and Persat, 2020). Embedded in the low-strength EPS, bacteria could grow in all directions, while in the high-strength EPS, bacteria prefer growing in the direction with the least resistance (Fig. 5E-H) (Zhang et al., 2021b; Liu et al., 2022). In R_GS_, the shear force on lateral sides of AnGS was higher due to the external gas supply (Fig. 5D), which peeled off loose structure on granular surface and slimmed the AnGS (Chen et al., 2007). Also, enhanced shear stimulated more EPS secretion, strengthening AnGS network structure and constraining lateral expansion. Notably, the shear force on granular upside was lower than other sides, leading to a lower EPS content and a weaker EPS strength. Moreover, hydraulic pressure on granular upside was the lowest (Fig. 5C). As bacteria preferred growing in the direction with less resistance, AnGS tended to extend along the upward direction and led to formation of ellipsoid-like AnGS. Considering that the enhanced flow shear is the key to cultivate ellipsoid-like granules, in practical applications, the produced gas from the reactor can be introduced back into the bottom of reactor. Also, the reactor can be designed with a larger ratio of height to diameter. These strategies can generate a larger upflow velocity, thereby enhancing the flow shear.

### 3.2 Advantages of ellipsoid-like AnGS in applications

The NRR and SAA of ellipsoid-like AnGS reached 16.3 ± 0.5 kg-N/(m^3^·d) and 478.3 ± 23.6 mg-N/(g-VSS·d), respectively, which were 22.6 % and 29.0 % higher than those of traditional sphere-like AnGS. Notably, previous studies mainly focused on size when considering morphological effect on AnGS performance (Ran et al., 2022). However, we showed granular shape was a previously overlooked factor affecting anammox performance.

In this study, the substrate permeation depth in sphere-like and ellipsoid-like AnGS is relatively constant due to the similar substrate concentration in bulk solution and mass transfer coefficient. However, functional bacteria can only play their roles in substrate permeable zone (van den Berg et al., 2021). With the increase of size, the fraction of substrate impermeable zone rose, while the overall mass transfer of AnGS deteriorated. Specifically, the mass transfer restriction of AnGS increased sharply when the size was over 2 mm (Xu et al., 2021b). The interior anammox bacteria would experience substrate starvation and even die due to limited substrate availability. In this study, the mass transfer efficiency declined with the increased size of sphere-like AnGS and the SAA decreased by 15.1 % at the end of phase I.

In addition to the size, the granular shape also plays an unexpected role in mass transfer. The proportion of substrate permeable zone of ellipsoid-like AnGS was significantly higher than that of sphere-like AnGS with the same granular volume (Fig. 5I and J). The proportion of substrate permeable zone of ellipsoid-like AnGS in R_GS_ can be elevated by over 30 % compared with that of sphere-like AnGS in R_CK_ (Text S1), contributing to better mass transfer and supporting more anammox bacteria. And the increased EPS secretion and nitrate production rate from anammox bacteria may be responsible for the increase of *Denitratisoma* abundance. Overall, the relatively smaller size and unique shape of ellipsoid-like AnGS jointly resulted in enhanced mass transfer, which explains the larger abundance of anammox bacteria (+12.2 %) and the higher SAA (+29.0 %) observed (Fig. 3A and S6).

## 4 Conclusions

In this study, a novel type of AnGS in a unique ellipsoid-like shape was obtained in the reactor with enhanced shear force. By systematically comparing the properties of traditional sphere-like and novel ellipsoid-like AnGS, we demonstrated that:

• The ellipsoid-like AnGS presented a higher specific anammox activity by 29.0 % and maximal nitrogen removal capacity by 22.6 %, compared to sphere-like AnGS.
• The ellipsoid-like AnGS exhibited a significantly smaller aspect ratio (-25.1 %) and granular size (-11.8 %), a higher EPS content and strength, as well as an enhanced mass transfer capacity due to the larger permeable zone. Also, the anammox bacterial abundance was 12.2 % higher in ellipsoid-like AnGS than in sphere-like AnGS. All these characteristics of ellipsoid-like AnGS jointly supported their superior capacity.
• The enhanced flow shear on AnGS side surface likely drove the formation of novel ellipsoid-like AnGS. The higher shear force on the side surface led to an increase of EPS (especially hydrophobic protein) and elastic modulus, thus constraining lateral expansion.

## 5 Materials and methods

### 5.1 Anammox bioreactors and long-term operation

Two lab-scale anammox reactors were operated in this study. R_GS_ was the experimental reactor with the external dinitrogen gas supply to enhance the flow shear (Fig. S7), while R_CK_ was the control reactor without any external gas supply (Fig. S8). Each bioreactor was made of a plexiglass column with a working volume of 1.0 L. The internal diameter and the ratio of height to diameter were 4 cm and 20 in the reaction zone, respectively. The synthetic wastewater was pumped into the distribution zone continuously, which went upwards through the reaction zone. The installed three-phase separator in the settling zone can separate the sludge from the mixed liquid, releasing supernatant and gas. A fraction of the supernatant was recycled from the center of the settling zone to the distribution zone at a fixed recirculation ratio of 7.5. External gas was continuously supplied through a gas distributor installed at the bottom of the reactor. The reactor was operated at 33 ± 2 °C. The influent pH was controlled at 7.5 ± 0.2. In Phase I, the operating parameters of the two reactors were the same except for the external gas supply. The surface gas flow rate of R_GS_ was elevated gradually with the gas velocity increasing from 0.33 to 1.33 cm/min (Table 1). In Phase II, the surface gas flow rate of R_GS_ was fixed at 0.75 L/h with the gas velocity of 1.00 cm/min, while the HRT of both reactors were decreased gradually to examine the nitrogen removal capacity.

**Table 1:** Operating conditions of R_CK_ and R_GS_ in different phases.

		Operation (d)	HRT (h)	Liquid upflow velocity (cm/min)	Gas flow rate (L/h)	Gas velocity (cm/min)
**Phase I**	**R_CK_**	1–119	1.5	7.48	0	0
	**R_GS_**	1–25	1.5	7.48	0.25	0.33
		26–72	1.5	7.48	0.50	0.67
		73–99	1.5	7.48	0.75	1.00
		100–105	1.5	7.48	1.00	1.33
		106–119	1.5	7.48	0.75	1.00
**Phase II**	**R_CK_**	120–137	1.3	8.63	0	0
		138–156	1.1	10.20	0	0
		157–169	1.0	11.22	0	0
		170–190	1.3	8.63	0	0
		191–226	1.1	10.20	0	0
	**R_GS_**	120–137	1.3	8.63	0.75	1.00
		138–156	1.1	10.20	0.75	1.00
		157–179	1.0	11.22	0.75	1.00
		180–226	0.9	12.47	0.75	1.00

**Note:** The Liquid upflow velocity referred to the velocity of the influent and reflux waterflow in the reactor, excluding the tail waterflow caused by the gas buoyancy.

### 5.2 Seeding sludge and influent compositions

The seeding sludge was collected from a parent bioreactor that had been run for more than 3 years. The NLR (nitrogen loading rate) and NRR (nitrogen removal rate) of the parent bioreactor were 9.93 ± 0.52 and 8.89 ± 0.48 kg-N/(m^3^·d), respectively. The AnGS with the size of 1–2 mm was screened out for the inoculation of R_CK_ and R_GS_. Synthetic wastewater was prepared according to Table S2. Ammonium-N and nitrite-N were supplied in the form of (NH_4_)_2_SO_4_ and NaNO_2_ with concentrations of 280 and 336 mg-N/L, respectively.

### 5.3 SAA determination

The SAA of AnGS was determined by batch experiments (Xu et al., 2022). About 1.0 g AnGS was collected and washed with phosphate buffer (0.1 M, pH=7.0) for 3 times. The washed AnGS and 50 mL basic solution (Table S2) were added into a 75 mL serum bottle. Subsequently, a mixture of carbon dioxide and argon was used to sparge the serum system for 10 min to remove oxygen in the liquid and headspace. The initial ammonium and nitrite concentrations were set at 50 and 60 mg-N/L, respectively. The tests were performed in a shaker with a speed of 150 rpm at 35 °C. Samples of 0.5 mL were collected hourly to test the concentrations of nitrogen profiles. The SAA was then calculated by summing the removal rates of ammonium and nitrite per gram of volatile suspended solids (VSS). The experiments were conducted in triplicate.

### 5.4 Granular size and shape determination

The size and aspect ratio of AnGS were determined by the QICPIC dynamic image system (Sympatec GmbH, Germany). About 5 mL AnGS was taken from the bioreactor, dispersed by a stirrer and then transported to the flow cell. The flowing AnGS was immediately projected by a fixed laser to obtain projection pictures. The measured granular size referred to the diameter of a circle of equal projection area (EQPC). The shape of AnGS was presented by the aspect ratio, which refers to the ratio of the minimum and maximum radial lengths of the projection area. The number of tested AnGS for each analysis was more than 1000 to ensure statistical robustness and reliability. All the tests were conducted in triplicate.

### 5.5 Mass transfer efficiency determination

Mass transfer efficiency *η*, defined as the ratio of the actual reaction rate (*r_a_*) to the maximum reaction rate (*r_p_*_)_, is used to characterize mass transfer capacity of AnGS (Xu et al., 2021a). In detail, *r_a_* is reflected by the SAA; And for a specific anammox bacteria, *r_p_* is positively related to the anammox bacterial abundance. Thus, the relative mass transfer efficiency of AnGS in different phases can be normalized based on the determined SAA and anammox bacterial abundance. The details were shown in Text S2.

### 5.6 Metabolomic profiling and quantitation analysis

About 2.0 g AnGS was collected and washed with phosphate buffer (0.1 mol/L, pH=7.0) for 3 times. Then the AnGS was broken and crushed by a glass grinder, followed by vibrational agitation using an oscillator to obtain a homogeneous bacterial suspension. This suspension was then resuspended in 5 mL ultrapure water at 4 °C. To facilitate metabolite extraction, the mixture was sonicated with a probe tip sonicator on ice, with parameters set to a pulse duration of 3 s with intervals between pulses, totaling 30 min of sonication at 30 % of maximum intensity (SCIENTZ-IID, 650W). Then 6 mL prechilled methanol/water (4:1, v/v) was added to the samples, which were incubated at -80 °C for 2 h to precipitate the cellular protein. After centrifugation (20 min at 10,000 g at 4 °C), the supernatants were collected and dried under a gentle steam of nitrogen gas at room temperature. The processed samples were stored in a -80 °C freezer. Metabolites including intracellular and extracellular compounds were then determined by high-performance liquid chromatography with tandem mass spectrometry (HPLC-MS). The untargeted metabolomic analysis was used for metabolome analysis (Zhao et al., 2018). All tests were performed in biological triplicate.

### 5.7 Other analysis

The concentrations of ammonium, nitrite, nitrate and VSS were all determined using the standard methods (APHA, 2017). The elastic modulus (Young's modulus) of AnGS was determined using an atomic force microscope (AFM) (Wang et al., 2022). DNA extraction, qPCR and Illumina high-throughput sequencing were all conducted according to Xu et al. (2021a). The raw sequencing reads of bacterial communities have been deposited into the NCBI Sequence Read Archive (SRA) under the accession number PRJNA1129192. The extraction and determination of EPS and amino acids in protein were performed by following Xu et al. (2022). The AnGS morphology was observed and captured by a stereoscope discovery V8 (Zeiss, Germany). The microstructure of AnGS was observed by transmission electron microscope (TEM). The fluid field around the AnGS was simulated by ANSYS FLUENT 15.0 software using the Eulerian model. In the fluid field simulation, the aspect ratio of simulated granules was set at 0.8 with a length of long axis of 2 mm, which was based on the determination results. And the flow velocity was set at 1.5 cm/s, which was in reference to our previous research results (Chen et al., 2024).

### 5.8 Statistical analysis

The error bars in this study represented the standard deviation from the three biological replicates. The statistical significance of the data between different experimental groups was determined with one-way analysis of variance. Any differences with *p* < 0.05 were considered statistically significant. The SPSS 22.0 software was employed for all statistical analyses.

## CRediT authorship contribution statement

**Dongdong Xu:** Writing – review & editing, Writing – original draft, Methodology, Conceptualization. **Tao Liu:** Writing – review & editing. **Chao Pan:** Methodology, Investigation. **Leiyan Guo:** Investigation, Data curation. **Jianhua Guo:** Writing – review & editing, Supervision. **Ping Zheng:** Supervision, Project administration. **Meng Zhang:** Writing – review & editing, Supervision, Funding acquisition.

## Declaration of competing interest

The authors declare that they have no known competing financial interests or personal relationships that could have appeared to influence the work reported in this paper.

## Data Availability

Data will be made available on request.

## Appendix Supplementary materials

Image, application 1
