# Supplementary material for: Novel ellipsoid-like granules exhibit enhanced anammox performance compared to sphere-like granules
Source: Water Res X. 2024 Oct 30;25:100270. doi: 10.1016/j.wroa.2024.100270 (PMC11550336; doi:10.1016/j.wroa.2024.100270)
Supplement: Supplementary file 1 [file mmc1.docx]

Supporting Information

**Novel ellipsoid-like granules exhibit enhanced anammox performance compared to sphere-like granules**

Dongdong Xu ^a, b^, Tao Liu ^b^, Chao Pan ^a^, Leiyan Guo ^a^, Jianhua Guo ^b *^, Ping Zheng ^a, c^, Meng Zhang ^a, c, d *^

^a^ *Department of Environmental Engineering,* *College of Environmental & Resource Sciences, Zhejiang University, Hangzhou, China*

^b^ *Australian Centre for Water and Environmental Biotechnology (ACWEB, formerly AWMC), The University of Queensland, St. Lucia, Queensland 4072, Australia*

^c^ *Zhejiang Province Key Laboratory for Water Pollution Control and Environmental Safety, Hangzhou, China*

^d^ *Innovation Center of Yangtze River Delta, Zhejiang University, Jiashan 314100, China*

^*^ Corresponding author: Meng Zhang; Jianhua Guo

Tel: +86-0571-88982819; +61-733463222

E-mail address: [zhangm_environment@zju.edu.cn](mailto:zhangm_environment@zju.edu.cn); jianhua.guo@uq.edu.au

**Pages: 11**

**Figures: 7**

**Tables: 2**

**Text S1**

**Calculation of the proportion of substrate permeable zone in AnGS**

The size parameters (*a*, *b* and *r*) of the granular sludge can be obtained according to the measured volumetric average diameter and the aspect ratio of AnGS, and the permeation distance (*l*) can be obtained according to the bulk substrate concentration and the mass transfer coefficient of AnGS. Furthermore, the proportion of permeation zone can be calculated.


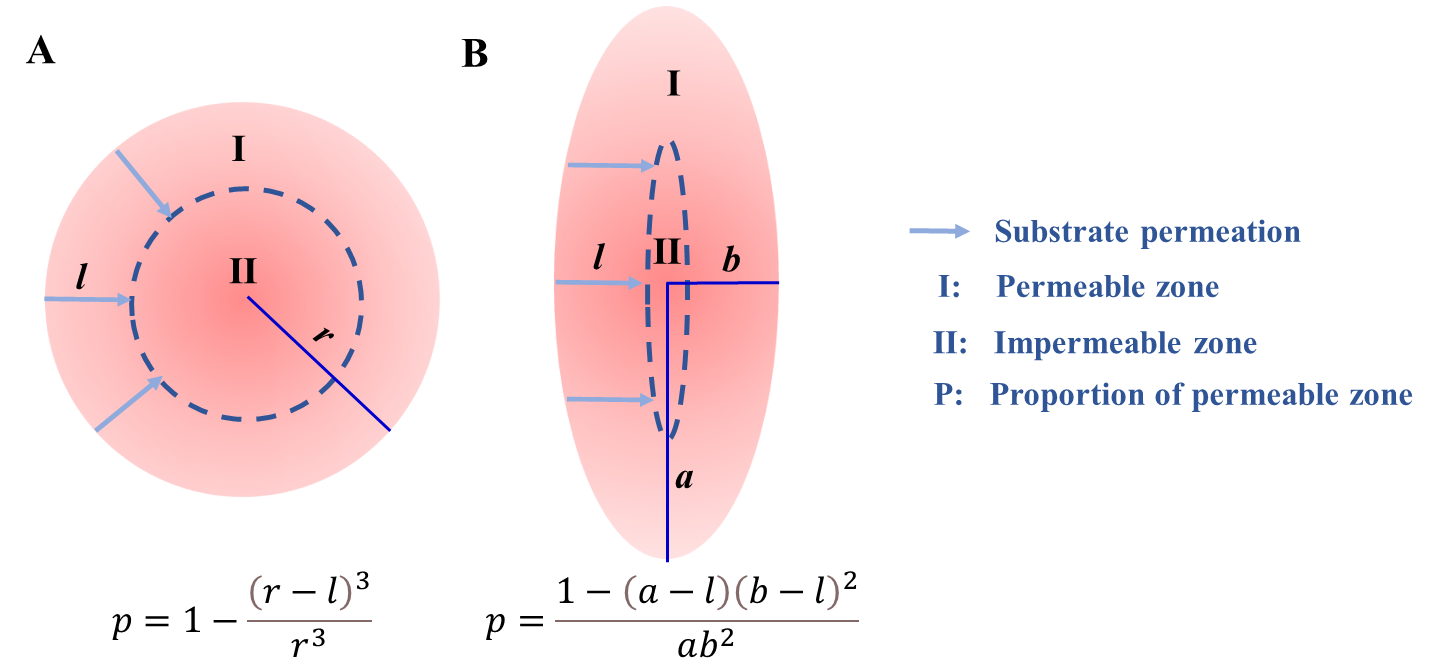


**Text S2**

**Calculation of the mass transfer efficiency**

Mass transfer efficiency factor $\eta$ was characterized as the ratio of the actual reaction rate (*r_a_*) to the maximum reaction rate without the mass transfer resistance (*r_p_*) [1]. *r_a_* can be reflected by the specific anammox activity [2], and *r_p_* is related to the anammox bacterial species and abundance [3]. For a specific anammox bacteria, *r_max_* and *K_S_* were constant. Hence, the relative mass transfer efficiency factor of AnGS in different phases can be normalized and compared following the equation [4].

$\eta=\frac{r_{a}}{r_{p}}$ [1]

$r_{a}=SAA\times\rho A$ [2]

$r_{p}=X\frac{r_{max}S}{K_{S}+S}=\rho AB\frac{r_{max}S}{K_{S}+S}$ [3]

$\eta=SAA/(B\frac{r_{max}S}{K_{S}+S})$ [4]

Where, SAA: The specific anammox activity, mg-N/(g-VSS⋅d); *ρ*: The density of AnGS, g-wet sludge/cm^3^; A: The biomass content, g-VSS/g-wet sludge; B: The abundance of anammox bacteria cells, copies/g-VSS; *r_max_*: The maximum substrate removal rate, mg-N/(copies⋅d); *K_S_*: The half-saturation constant, mg-N/L; *S*: Substrate concentration, mg-N/L.

**Fig. S1** Variations of the AnGS’s size distribution in R_CK_ (A) and R_GS_ (B).

**Fig. S2** VSS concentrations of R_CK_ and R_GS_ during operation.


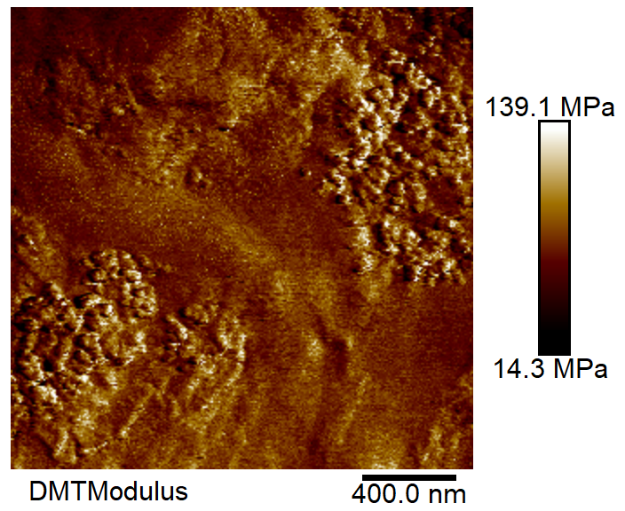

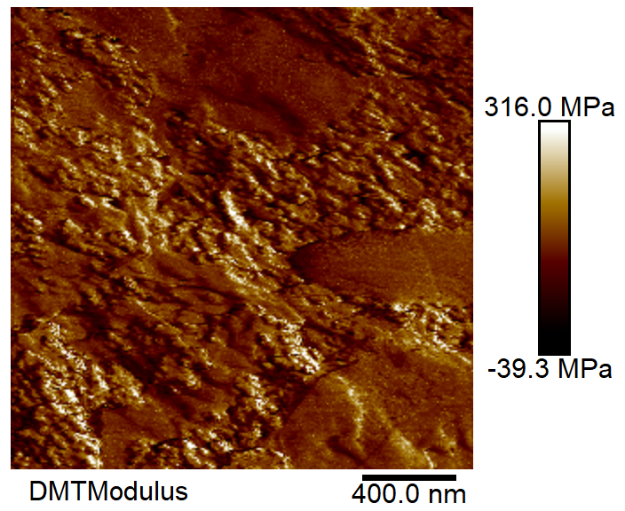


**A**

**B**

**Fig. S3** Elasticity modulus of lateral sides (A) and upside (B) of AnGS in R_GS_ on day 220.

**Fig. S4** EPS content of lateral sides (A) and upside (B) of AnGS in R_GS_ on day 220.

**Fig. S5** The contents of hydrophobic amino acids in protein of EPS in R_GS_ and R_CK_ on day 220.

**Fig. S6** Microbial community of AnGS in R_CK_ and R_GS_ at genus level during operation.


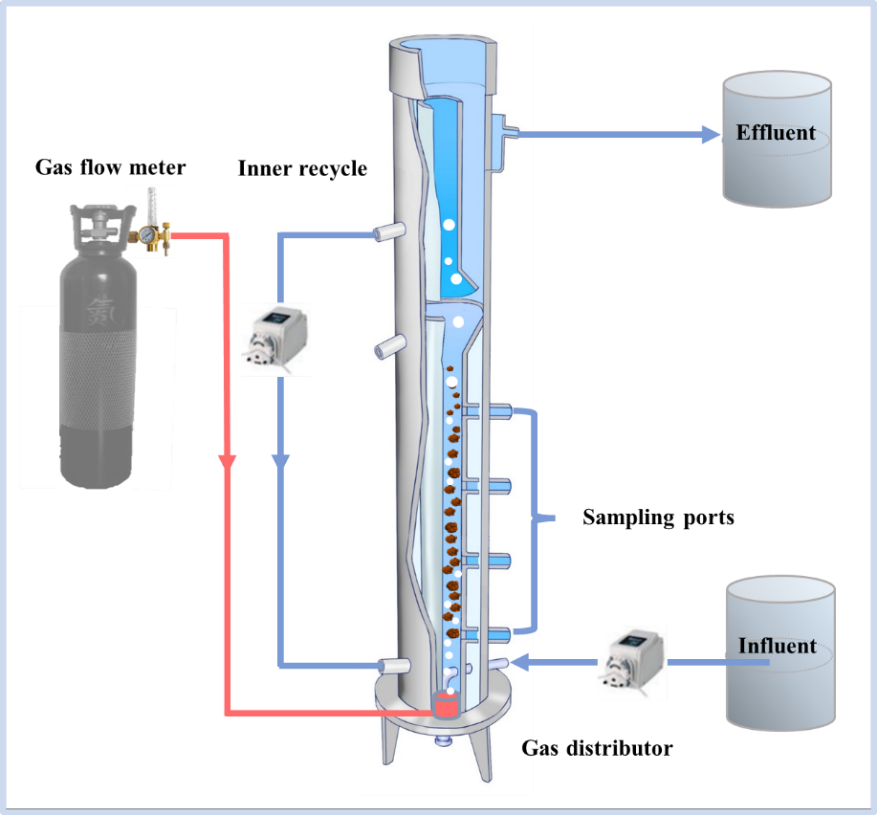


**Fig. S7** Schematic of anammox reactor with the external gas supply (R_GS_).


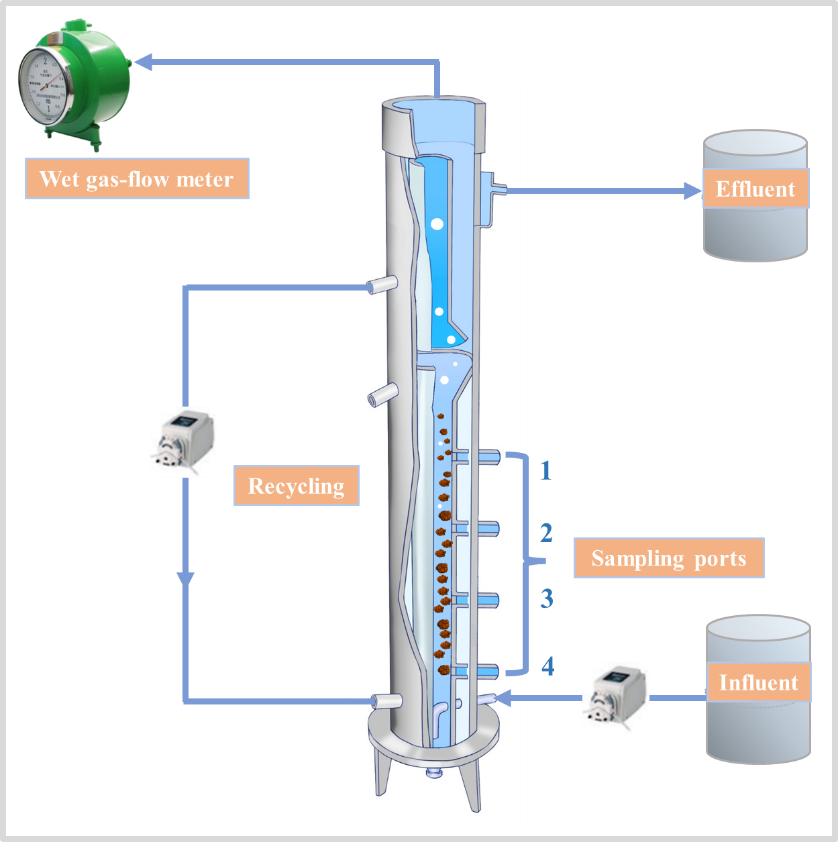


**Fig. S8** Schematic diagram of the control anammox granular sludge bed reactor (R_CK_).

**Table S1** Short and full names of amino acids

| Short name | Full name | Short name | Full name |
| --- | --- | --- | --- |
| Ala | Alanine | Asp | Asparatic acid |
| Cys | Cystein | Thr | Threonine |
| Tyr | Tyrosine | Ser | Serine |
| Met | Methionine | Glu | Glutamic acid |
| Ile | Isoleucine | Gly | Glycine |
| Leu | Leucine | His | Histidine |
| Val | Valine | Lys | Lysine |
| Phe | Phenylalanine | Arg | Arginine |
| Pro | Proline | Asn | Asparagine |
| Trp | Tryptophan | Gln | Glutamine |

**Table S2** Composition of the basic solution

| Composition | Concentration(g/L) | Composition | Concentration(mg/L) |
| --- | --- | --- | --- |
| KHCO_3_ | 0.24 | EDTA-2Na | 15 |
| NaHCO_3_ | 0.8 | H_3_BO_4_ | 0.014 |
| MgSO_4_·7H_2_O | 0.3 | MnCl_2_·4H_2_O | 0.99 |
| KH_2_PO_4_ | 0.0175 | CuSO_4_·5H_2_O | 0.25 |
| CaCl_2_ | 0.0175 | ZnSO_4_·7H_2_O | 0.43 |
|  |  | NiCl_2_·6H_2_O | 0.19 |
|  |  | NaMoO_4_ | 0.22 |
| - | - | CoCl_2_·6H_2_O | 0.24 |
|  |  | EDTA-2Na | 5 |
|  |  | FeSO_4_·7H_2_O | 5 |
